# Draft Genome Sequences of 11 *Lactococcus lactis* subsp. *cremoris* Strains

**DOI:** 10.1128/genomeA.01739-16

**Published:** 2017-03-16

**Authors:** Michiel Wels, Lennart Backus, Jos Boekhorst, Annereinou Dijkstra, Marke Beerthuyzen, Roland J. Siezen, Herwig Bachmann, Sacha A. F. T. van Hijum

**Affiliations:** a Kluyver Centre for Industrial Fermentations, NIZO, Ede, The Netherlands; b Centre for Molecular and Biomolecular Informatics, Radboud Institute for Molecular Life Sciences, Radboud umc, Nijmegen, The Netherlands; c TI Food and Nutrition, Wageningen, The Netherlands; d Microbial Bioinformatics, Ede, The Netherlands; e Faculty of Earth and Life Sciences, Systems Bioinformatics, VU University Amsterdam, Amsterdam, The Netherlands

## Abstract

The lactic acid bacterium *Lactococcus lactis* is widely used for the fermentation of dairy products. Here, we present the draft genome sequences of 11 *L. lactis* subsp. *cremoris* strains isolated from different environments.

## GENOME ANNOUNCEMENT

*Lactococcus lactis* is a Gram-positive bacterium that is predominantly found on plant material and in the dairy environment (1, 2). It is extensively used in dairy fermentations (3), which is mainly due to its role in the development of texture and flavor through, e.g., proteolysis and the production of volatile flavor compounds (4). It also contributes to food preservation through the production of organic acids and bacteriocins, such as nisin (5). Four *L. lactis* subspecies have been defined: *L. lactis* subsp. *lactis* (6), *L. lactis* subsp. *cremoris* (7), *L. lactis* subsp. *hordniae* (8), and *L. lactis* subsp. *tructae* (9). In this study, we report the draft genome sequences of 11 *L. lactis* subsp. *cremoris* strains (Table 1).

**TABLE 1:** Overview of the 11 *L. lactis* subsp. *cremoris* strains in NCBI BioProject PRJNA286840

Strain	Accession no.	Isolation source (reference)
AM2	LITE00000000	Dairy starter (12)
B40	LITC00000000	Scandinavian ropy milk (13)
FG2	LITD00000000	Dairy starter (12)
HP	LIYE00000000	Dairy starter (12)
KW10	LIYF00000000	Kaanga wai (12)
LMG6897	LISZ00000000	Cheese starter (12)
NCDO763	LITG00000000	Dairy starter (12)
N41	LITA00000000	Soil and grass (12)
P7266	LITB00000000	Litter on pastures (12)
SK110	LITF00000000	Dairy starter (12)
V4	LIYG00000000	Raw sheep milk (12)

The strains used in this study were grown overnight in 5 ml of GM17 broth at 30°C. After propagation in fresh medium, cells were harvested during exponential growth, and cell pellets were resuspended in a buffer (6.7% sucrose, 1 mM EDTA, 50 mM Tris-HCl [pH 8.0]) and incubated with RNase (0.5 mg/ml) and lysozyme (2 mg/ml) at 37°C for 1 h. Subsequently, the samples were treated with SDS (1% [wt/vol] final concentration) at 37°C for 10 min. The total DNA was extracted by phenol-chloroform, precipitated with isopropanol and sodium acetate, and dissolved in sterile water (10).

The whole-genome sequencing was performed at GATC Biotech (Konstanz, Germany) with 50-bp paired-end libraries on an Illumina HiSeq 2000. Raw sequence reads of each of the genomes were assembled *de novo* using IDBA (11), with default parameters, at a target coverage of 50×. This resulted in draft genomic sequences for 11 *L. lactis* subsp. *cremoris* strains (Table 1). Annotation of the contig sequences was performed by the RAST server (14).

### Accession number(s).

The genome sequences of the 11 *L. lactis* subsp. *cremoris* strains have been deposited as whole-genome shotgun projects at DDBJ/EMBL/Genbank under the accession numbers listed in Table 1.
